# Chemical Trends of the Bulk and Surface Termination-Dependent Electronic Structure of Metal-Intercalated Transition Metal Dichalcogenides

**DOI:** 10.1021/acs.chemmater.4c00824

**Published:** 2024-07-23

**Authors:** Brendan Edwards, Darius-A. Deaconu, Philip A. E. Murgatroyd, Sebastian Buchberger, Tommaso Antonelli, Daniel Halliday, Gesa-R. Siemann, Andela Zivanovic, Liam Trzaska, Akhil Rajan, Edgar Abarca Morales, Daniel A. Mayoh, Amelia E. Hall, Rodion V. Belosludov, Matthew D. Watson, Timur K. Kim, Deepnarayan Biswas, Tien-Lin Lee, Craig M. Polley, Dina Carbone, Mats Leandersson, Geetha Balakrishnan, Mohammad Saeed Bahramy, Phil D. C. King

**Affiliations:** † SUPA, School of Physics and Astronomy, University of St Andrews, St Andrews KY16 9SS, U.K.; ‡ Department of Physics and Astronomy, University of Manchester, Oxford Road, Manchester M13 9PL, U.K.; § Max Planck Institute for Chemical Physics of Solids, Nöthnitzer Strasse 40, Dresden 01187, Germany; ∥ Diamond Light Source Ltd, Harwell Science and Innovation Campus, Didcot OX11 0DE, U.K.; ⊥ Department of Physics, University of Warwick, Coventry CV4 7AL, U.K.; # Institute for Materials Research, Tohoku University, Sendai 980-08577, Japan; ∇ MAX IV Laboratory, Lund University, P.O. Box 118, Lund 221 00, Sweden

## Abstract

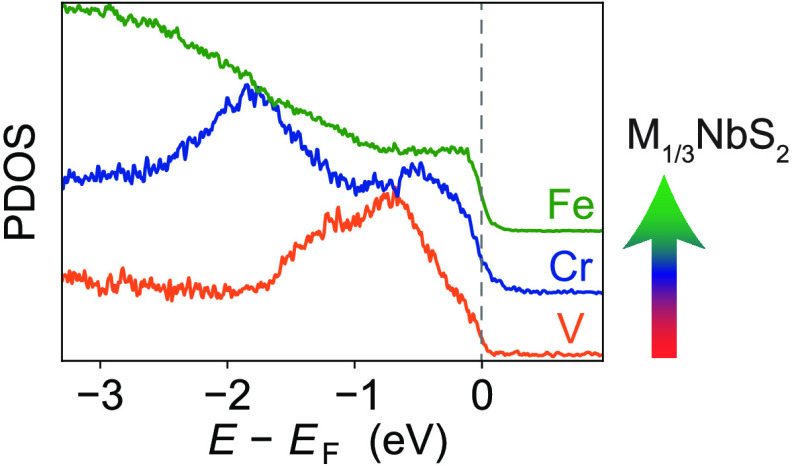

The addition of metal intercalants into the van der Waals gaps of transition metal dichalcogenides has shown great promise as a method for controlling their functional properties. For example, chiral helimagnetic states, current-induced magnetization switching, and a giant valley-Zeeman effect have all been demonstrated, generating significant renewed interest in this materials family. Here, we present a combined photoemission and density-functional theory study of three such compounds: , , and , to investigate chemical trends of the intercalant species on their bulk and surface electronic structure. Our resonant photoemission measurements indicate increased hybridization with the itinerant NbS_2_-derived conduction states with increasing atomic number of the intercalant, leading to pronounced mixing of the nominally localized intercalant states at the Fermi level. Using spatially and angle-resolved photoemission spectroscopy, we show how this impacts surface-termination-dependent charge transfers and leads to the formation of new dispersive states of mixed intercalant-Nb character at the Fermi level for the intercalant-terminated surfaces. This provides an explanation for the origin of anomalous states previously reported in this family of compounds and paves the way for tuning the nature of the magnetic interactions in these systems *via* control of the hybridization of the magnetic ions with the itinerant states.

## 1 Introduction

Transition metal dichalcogenides (TMDs) have become the subject of intensive research, owing to the diverse properties they host.^1−3^ These include metallic and insulating electronic structures,^4^ several notable examples of spin-valley locking,^5−9^ collective phenomena such as charge density waves and superconducting states,^10−14^ and a myriad of topological phases.^15−17^ Intercalating magnetic transition metal ions into the van der Waals gap of 2H-TMDs has emerged as a powerful method to further stabilize novel magnetic states and textures, where the intercalant species nominally act as local magnetic moments.^18,19^ At critical concentrations, they occupy periodic sites that break the centrosymmetry of the host compounds. This allows for the presence of antisymmetric Dzyaloshinskii–Moriya exchange interactions, which underpin the formation of a host of noncollinear magnetic orders.^20−22^

**Figure 1:**
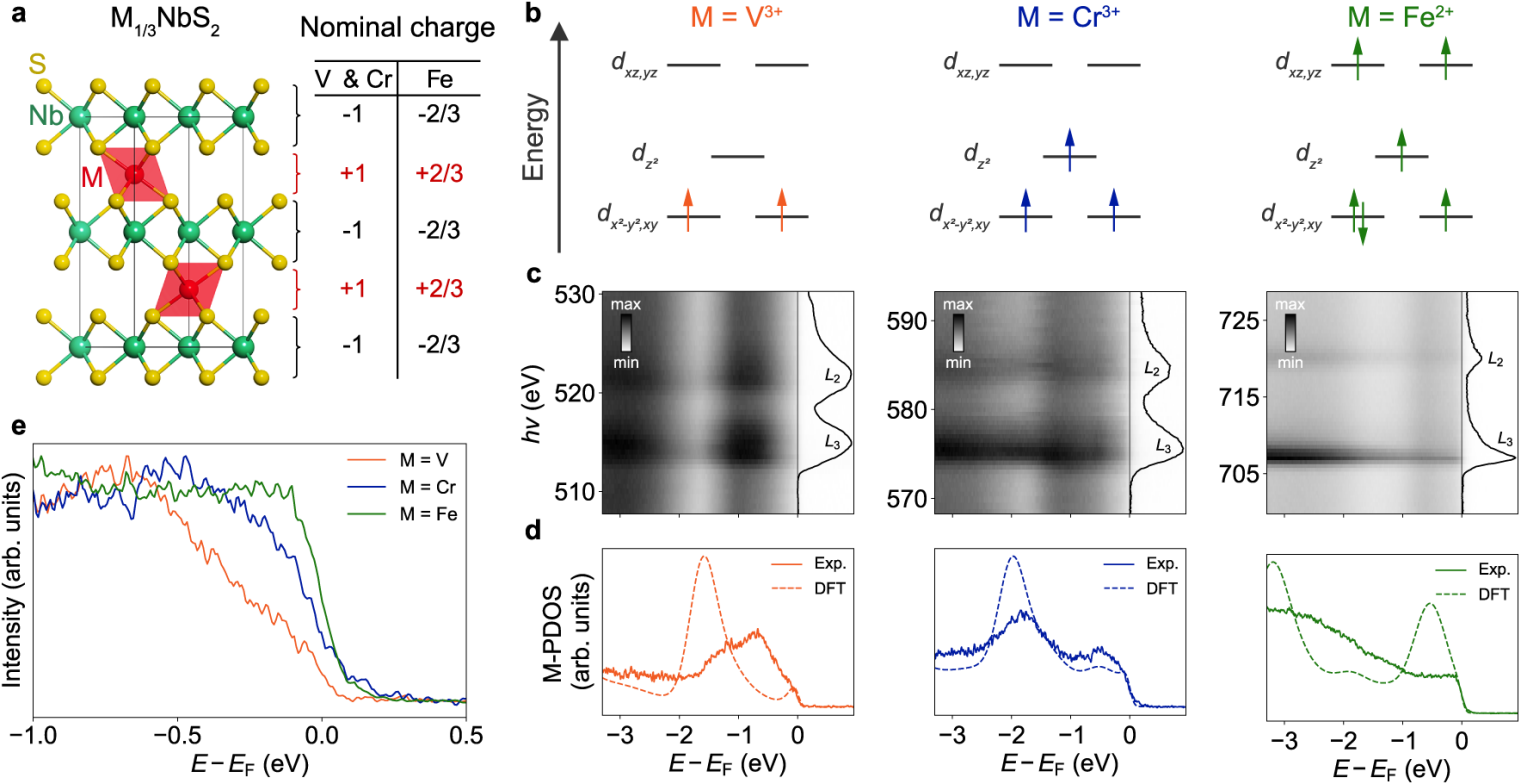
Electronic structure of  compounds. (a) Crystal structure displaying the intercalant-dependent electronic charge of layers described by a simple ionic picture. The intercalants are surrounded by S ligands in octahedral-like coordination environments, as indicated by shaded red regions. (b) Schematics illustrating the crystal field splitting and electron filling of the 3*d* intercalant orbitals in , , and , after ref (24). While V and Cr atoms possess +3 oxidation states, Fe atoms will exist in +2 oxidation states. (C) Angle-integrated ResPES spectra (*T* = 27 K, LH polarization) measured across the intercalant *L*_2,3_ absorption edges (shown in the XAS measurements on the right of the panels) of  and . (d) Experimental measures of the intercalant-derived PDOS of , , and , extracted from the difference between cuts of the on- and off-resonance spectra in (c). These are compared to the intercalant-derived PDOS calculated using DFT (see Section 2), for which a 200 meV broadening has been applied to simulate broadening from lifetime and experimental effects. The total areas under the curves have been normalized for clarity. (e) Direct comparison of the near Fermi level experimentally determined intercalant-derived PDOS shown in (d), normalized to the maximum measured intensity within the shown energy range. A trend of increasing intercalant-derived PDOS present at the Fermi level with increasing atomic number is observed across the materials studied.

Here, we investigate intercalated TMDs of the form , where M = (V, Cr, and Fe). At this critical composition, the intercalants occupy interstitial sites to form a superlattice, which generates a new periodic potential with a  periodicity.^23^ The resulting crystal structure is shown in Figure 1a, where it can be seen that the intercalants form distorted octahedral coordination environments with the surrounding S ligands.^24^ The distance between adjacent intercalants is too large for significant direct exchange interactions. Instead, the magnetism must be mediated through the NbS_2_ layers, and thus the dominant exchange interaction in these compounds is frequently thought to be a Ruderman–Kittel–Kasuya–Yosida (RKKY) mechanism.^18^ In this scenario, the modulation of the NbS_2_ electronic structure that results from the intercalation is described by a rigid band shift picture, where there is an ionic charge transfer of electrons from intercalant 3*d* and 4*s* orbitals into the Nb 4*d* orbitals with minimal hybridization between the intercalant- and Nb-derived states, leaving the electronic structure otherwise unperturbed. However, evidence of stronger hybridizations between intercalant-derived local moment and Nb-derived itinerant states was reported in  and ,^25−28^ indicating that the simplest RKKY picture may not apply to all compounds in this material class, and pointing toward the choice of intercalant species playing a pivotal role in the electronic structure and nature of the magnetic interactions.

This is reflected in the disparate magnetic structures that are observed. In , the local V moments exhibit an A-type antiferromagnetic order, with an additional small canting of spins along the out-of-plane direction, which is reported to lead to a net uncompensated ferromagnetic moment.^22,29^ instead hosts a chiral helimagnetic ground state,^20,30,31^ where a topologically protected chiral soliton lattice can be formed and modulated by an external magnetic field.^21,32,33^ In , the Fe sublattice orders antiferromagnetically,^34^ with signatures of three-state nematicity,^35^ and it has been shown to support current-induced magnetization switching at surprisingly low current densities.^36^ Moreover, while the bulk magnetic properties of these compounds are well-studied, the magnetic phenomena at their termination-dependent surfaces—where markedly distinct charge transfer occurs—requires further exploration, as evident, for example, by the recent demonstration of a giant valley-Zeeman coupling in the NbS_2_ surface layer of .^37^

Here, we report a systematic study of the bulk and surface electronic structure of , , and  by combining resonant photoemission spectroscopy (ResPES) and microscopic area spatially and angle-resolved photoemission spectroscopy (μ-ARPES) with density-functional theory (DFT) calculations. Our results demonstrate that intercalant-derived spectral weight is absent at the Fermi level of  but increasingly develops upon moving to the Cr- and Fe-based compounds, indicating the breakdown of a pure RKKY-like exchange mechanism. We show how these varying levels of hybridization lead to contrasting features in the surface-termination dependent electronic structures, with chemical potential variations that decrease with the increasing atomic number of the intercalant. Our results additionally allow us to identify the origin of the anomalous states reported at the Fermi level in this family of compounds,^25,28,38−40^ as the result of hybridizations between states derived from the electron-doped intercalant-terminated surface and subsurface NbS_2_ layer.

## 2 Methods

### 2.1 Crystal Growth

Single crystals of  (M = V, Cr, and Fe) were produced by the chemical vapor transport technique using iodine as the transport agent, as described previously.^22,31^ Presynthesized polycrystalline powders of the starting compositions, , along with ∼5 mg/cm^3^ of the transport agent, were placed in an evacuated and sealed quartz tube. The tube was then heated with one end held at 950^◦^C and the other at 800^◦^C (M = Cr) or 850^◦^C (M = V and Fe) for 3 weeks before cooling to room temperature. Crystals in the form of platelets ranging from ∼1 to 5 mm along their longest edges were formed. A backscattering X-ray Photonic-Science Laue camera system was used to assess the quality of the single crystals. Their composition was estimated by using energy-dispersive X-ray spectroscopy analysis in a scanning electron microscope. Single crystal X-ray diffraction and transmission electron microscopy were used to confirm the noncentrosymmetric *P*6_3_22 space group adopted by these crystals for the critical composition of .^22,31^ In addition, we have confirmed the stoichiometry *via* measurements of the magnetic susceptibility, which for the Fe-based compound (see Supplementary Figure 9), is extremely sensitive to the precise stoichiometry achieved.^34^

### 2.2 ResPES and XAS

Resonant photoemission spectroscopy (ResPES) and X-ray absorption spectroscopy (XAS) measurements were performed at the I09 beamline at Diamond Light Source using linear horizontal (LH) polarized light with photon energies between 507 and 729 eV and a beam spot size of  μm^2^. The samples were cooled to a base temperature of ∼27 K and cleaved *in situ* in a base pressure below 10^–10^ mbar. ResPES measurements were performed using a Scienta Omicron EW4000 analyzer, while XAS spectra were obtained from the sample drain currents in total electron yield mode. The partial density of states of the intercalant species was extracted by subtracting two photoemission spectra measured at photon energies on and just below the corresponding *L*_3_ edge.

### 2.3 μ-ARPES and XPS

Microscopic area spatially and angle-resolved photoemission spectroscopy (μ-ARPES) and X-ray photoelectron spectroscopy (XPS) measurements were performed at the nano-ARPES branch of the I05 beamline at Diamond Light Source using linearly polarized light with photon energies between 79 and 200 eV and a beam spot size of  μm^2^. The samples were mounted on a piezo-manipulator at a base temperature of ∼35 K, cleaved *in situ* in a base pressure below 10^–10^ mbar, and measured using a Scienta Omicron DA30 analyzer. Additionally, the measurements of  presented in Figure 3a,b and Supplementary Figure 4 were performed at the Bloch beamline at MAX IV Laboratory, at a base temperature of ∼20 K, using a beam spot size of  μm^2^.

### 2.4 DFT Calculations

The bulk electronic structure calculations were performed within density-functional theory (DFT) using the Perdew–Burke–Ernzerhof exchange–correlation functional^41^ and pseudopotential method as implemented in the Vienna Ab initio Simulation Package.^42,43^ Relativistic effects, including spin–orbit interactions, were fully taken into account. A  supercell containing six formula units of NbS_2_ and two M atoms occupying the  and  intercalation sites, as shown in Figure 1a, was used. The corresponding Brillouin zone was sampled using a *k*-mesh and plane-wave cutoff energy of 400 eV. For M = V (Cr and Fe), an antiferromagnetic (ferromagnetic) ordering along [001] was imposed. An additional on-site Hubbard term with an effective *U* value of  eV (1 eV) was added to the 3*d* orbitals of the V and Fe (Cr) to reproduce the experimentally observed energetic alignment of the corresponding M states. We note that larger values of *U* did not lead to adequate agreement with our experimental measurements, establishing how the strong screening environment from the NbS_2_ layers here leads to smaller *U* values than might otherwise be assumed for these 3*d* transition metals. Projected density of states calculations were performed by using a Wigner–Seitz radius of 1.3 Å for the intercalant ion and a Gaussian broadening factor of 200 meV to simulate the effect of lifetime broadening observed in the experiment. For the PDOS plots in Figure 1, a Fermi–Dirac distribution was also applied to exclude states above the Fermi level.

For the surface structure calculations, a slab containing six units of  stacked along the crystalline *c* axis with a vacuum space of 12 Å was constructed. The corresponding Brillouin zone was sampled using a *k*-mesh. All other parameters were kept the same as those in the bulk calculations. The excess and depletion charge distribution shown in Figure 4a,b was obtained by computing the charge distribution for the entire slab, then subtracting the individual charge densities from the NbS_2_ and Cr centers. The latter were obtained by removing Cr and NbS_2_ layers in the same slab, respectively. The remaining was treated as excess whenever this difference was positive, and as depletion whenever this difference was negative.

## 3 Results

To gain an initial understanding of the impact of the intercalant species on the electronic structures of the three compounds discussed here, we first consider an approximation of their electronic configurations using crystal field theory. The trigonally distorted octahedral environment of the intercalated transition metal ions would be expected to split their *d* orbital energy levels as shown schematically in Figure 1b.^24^ In  and , the intercalants are expected to have a nominal +3 oxidation state,^18^ leading to a net donation of one electron from the intercalant layers to the NbS_2_ layers (see Figure 1a). Two (three) electrons will thus fill the V (Cr) *d* orbital manifold in a high-spin configuration, as shown schematically in Figure 1b, due to the relatively weak crystal field.^24^ This simple picture is fully consistent with our bulk DFT calculations, where we find a local magnetic moment of 2 μ_B_ (3 μ_B_) per V (Cr) ion, as well as our ResPES and XAS measurements for these compounds, shown in Figure 1c. Extracting the intercalant-derived partial density of states (PDOS) from our ResPES measurements, as shown in Figure 1d (see also Supplementary Figure 6), we find a single broad PDOS contribution centered around 1 eV below the Fermi level for , which we attribute to the localized V-derived states nominally in the  orbitals. In comparison, a pronounced two-peak structure is observed in the Cr-derived PDOS, consistent with the higher *d* orbital population shown in Figure 1b (see also Supplementary Figure 5 for a comparison between the intercalant-projected and total density of states). While our calculations do not quantitatively capture all features of our experimental measurements, such as an energy-dependent broadening due to lifetime effects that are beyond DFT, the agreement of the core features allows us to confidently assign chemical trends in the electronic structure, as discussed below.

In contrast to the V and Cr case, previous reports^24^ indicate that  hosts the intercalated Fe ions in +2 oxidation states. This will lower the net charge transfer from the Fe to the NbS_2_ layers (Figure 1a) and will lead to a nominal  electron configuration of the Fe sites. The crystal field is still expected to be relatively weak,^24^ and so a high-spin configuration can still be expected, as shown in Figure 1b. Consistent with this, a broad distribution of Fe-derived PDOS is found throughout the valence band in both our ResPES measurements and our corresponding DFT calculations. Our calculations also reveal that each Fe effectively maintains a local magnetic moment of 3.6 μ_B_, further confirming its distinctive 2+ ionic state in this group of compounds. Interestingly, we find that the resulting Fe-derived spectral weight persists right up to the Fermi level, with the intercalant-derived PDOS at the Fermi level showing a systematic increase with an increasing atomic number of the intercalated transition metal in both our calculated and experimentally determined intercalant-derived PDOS (Figure 1d,e).

The simplest picture of the metal-intercalated TMDs assumes that the intercalant atoms act as local moments, otherwise modifying the electronic structure of NbS_2_ purely *via* electron doping in a rigid band shift picture,^18,19^ with the magnetic interactions described within an RKKY picture. While our measurements here show that this picture remains approximately valid for the V cations, it increasingly breaks down for Cr and Fe, instead pointing to a more complex interplay between the local moment and itinerant states (see also Supplementary Figure 8). Indeed, given that conduction in these compounds is largely governed by Nb 4*d*-derived orbitals, the presence of intercalant-derived weight at the Fermi level points to stronger hybridizations between local moment intercalant-derived and itinerant Nb-derived states. For the Fe-based compound, in particular, the strong intercalant-derived weight at the Fermi level and broad bandwidth of Fe-derived states points to a strong hybridization between the intercalant- and NbS_2_-derived states, leading to a collapse of the rigid band shift picture, similar to the conclusions recently drawn from studies of .^27,28^ The Cr-based material appears intermediate between these regimes,^25^ and we thus conclude that there is a strong and approximately monotonic chemical trend of increasing hybridization tied to the increasing atomic number of the intercalated metal species here, which can in turn be expected to dominate the magnetic interactions in these compounds.

To investigate these electronic structure trends in more detail, we performed *k*-resolved electronic structure measurements using ARPES. A significant complication, however, is that the interlayer charge transfer discussed above becomes strongly modified at the surface of such materials.^37,40,44,45^ ARPES, in particular, when performed at the photon energies used here, is a highly surface-sensitive technique. It is thus this distinct surface electronic structure that will be probed. Furthermore, due to the natural cleavage plane being situated between the intercalant and NbS_2_ layers, both intercalant-exposed and NbS_2_-exposed surface terminations can be expected. Indeed, such distinct terminations have previously been observed in STM measurements over a field of view of ∼100 nm.^38^ Moreover, spectromicroscopy has shown that such regions are randomly distributed across the surface, with characteristic variations observed at the few-micron scale.^37^ Additional factors relating to crystallographic defects, such as polycrystalline structures, stacking faults, or the presence of multiple polymorphs, would also lead to variations of the surface at the mesoscopic level. In the following, however, we consider that spatial variations in the surface terminations are the key differentiating feature.

**Figure 2:**
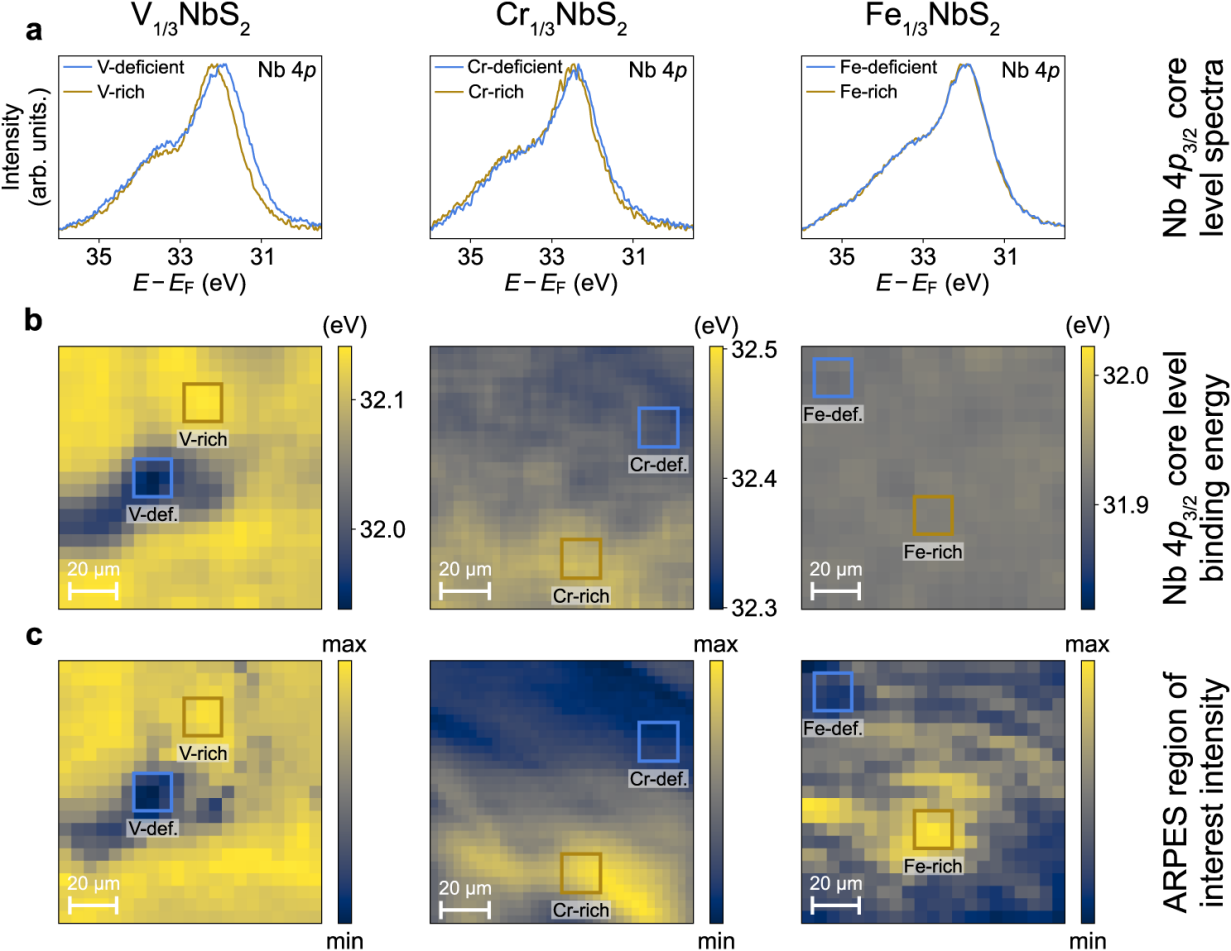
Spatial mapping of  compounds. (a) Representative Nb 4*p* core-level spectra ( eV, LH polarization) of the intercalant-deficient (blue) and intercalant-rich (brown) surface terminations of  (*T* = 32 K),  (*T* = 35 K), and  (*T* = 43 K), extracted from the regions of spatial mapping data indicated in (b). (b) Spatial maps displaying the fitted binding energies of the Nb 4 core levels of , , and . To allow for a comparison of the intercalant-dependent binding energy shifts, the maps were plotted using color scales with the same size binding energy range. (c) Spatial maps (LH polarization) of  (*T* = 33 K,  eV),  (*T* = 35 K,  eV), and  (*T* = 36 K,  eV) displaying the integrated spectral weight within the region of interest of ARPES measurements of the low-energy electronic structure corresponding to intercalant-derived states (see Supplementary Figure 1 for more information). High (yellow) and low (blue) intensity regions correspond to the intercalant-rich and intercalant-deficient surface terminations, respectively.

To enable probing of different surface terminations, we thus performed ARPES measurements using a focused light source (μ-ARPES, see Section 2). To identify the unique surface terminations of , , and , we initially performed spectromicroscopy measurements using photoemission of the Nb 4*p* core level (see Figure 2a). A 200 eV photon energy was used for these measurements, providing sufficient surface sensitivity to characterize the distinct surface terminations.

To analyze the spatial variations of the Nb 4*p* core level, we fit the peaks of the spectra across the spatial mapping region and show the spatial dependence of the Nb  binding energy in Figure 2b. Considering first the case of , we observe the emergence of distinct regions in the spatial map, with areas of decreased (increased) binding energy of the core level, with shifts visible on the order of 200 meV (see also Figure 2a). These correspond to chemical potential variations that result from decreased (increased) electron doping of the surface region. We assign the former regions to intercalant-deficient surface terminations hosting significant concentrations of exposed NbS_2_ surface layers and low concentrations of surface intercalants. Here, the absence of electron donation into the surface NbS_2_ layer from the missing intercalant atoms atop results in an effective hole-like self-doping of the surface layer. In contrast, we assign the more electron-doped regions to intercalant-rich surface terminations, where the previously intercalated atoms are now effectively adsorbed on the sample surface. The missing NbS_2_ layers above such a surface plane can no longer act as electron acceptors, leading to an electron-like self-doping of the intercalant-terminated surface layer.

While the distinct spatial regions are sharply defined for the V-based compound in Figure 2b, with corresponding pronounced changes in the core level spectra (Figure 2a), more subtle variations are observed across the surface of the Cr-based compound. The lack of variation becomes even more extreme in the Fe-based compound, where negligible contrast is observed beyond the experimental noise. Nonetheless, unique surface terminations can still also be reliably identified by performing spectromicroscopy of the low-energy electronic structure using ARPES, as shown in Figure 2c where the integrated spectral weight within the region of interest corresponding to intercalant-derived states associated with the intercalant-rich surface terminations is shown (see Supplementary Figures 1 and 2). We suggest that the reduced chemical potential variations seen in the Nb  binding energy spatial maps of  and  in Figure 2b are the result of increased hybridizations with the intercalant species in these compounds, as compared to  (as identified above). This would result in a reduced ionic charge transfer from the intercalant to the NbS_2_ layer, making the termination-dependent core level spectral changes less clear, while also leading to a stronger bonding between the intercalant and the NbS_2_ layers and thus producing a less natural cleavage plane in the material. We would therefore expect smaller areas of distinct surface termination and thus increased regions probed with mixed terminations, making the spatial variation less clear in the Cr- and Fe-based compounds, entirely consistent with our measurements in Figure 2. In this respect, we note that previous scanning tunneling microscopy studies have observed locally rather disordered terminations for the Cr-terminated surface of ,^38^ again supporting this picture. We therefore refer here to intercalant-rich and intercalant-deficient terminations rather than assigning these as pure and clean surface terminations.

**Figure 3:**
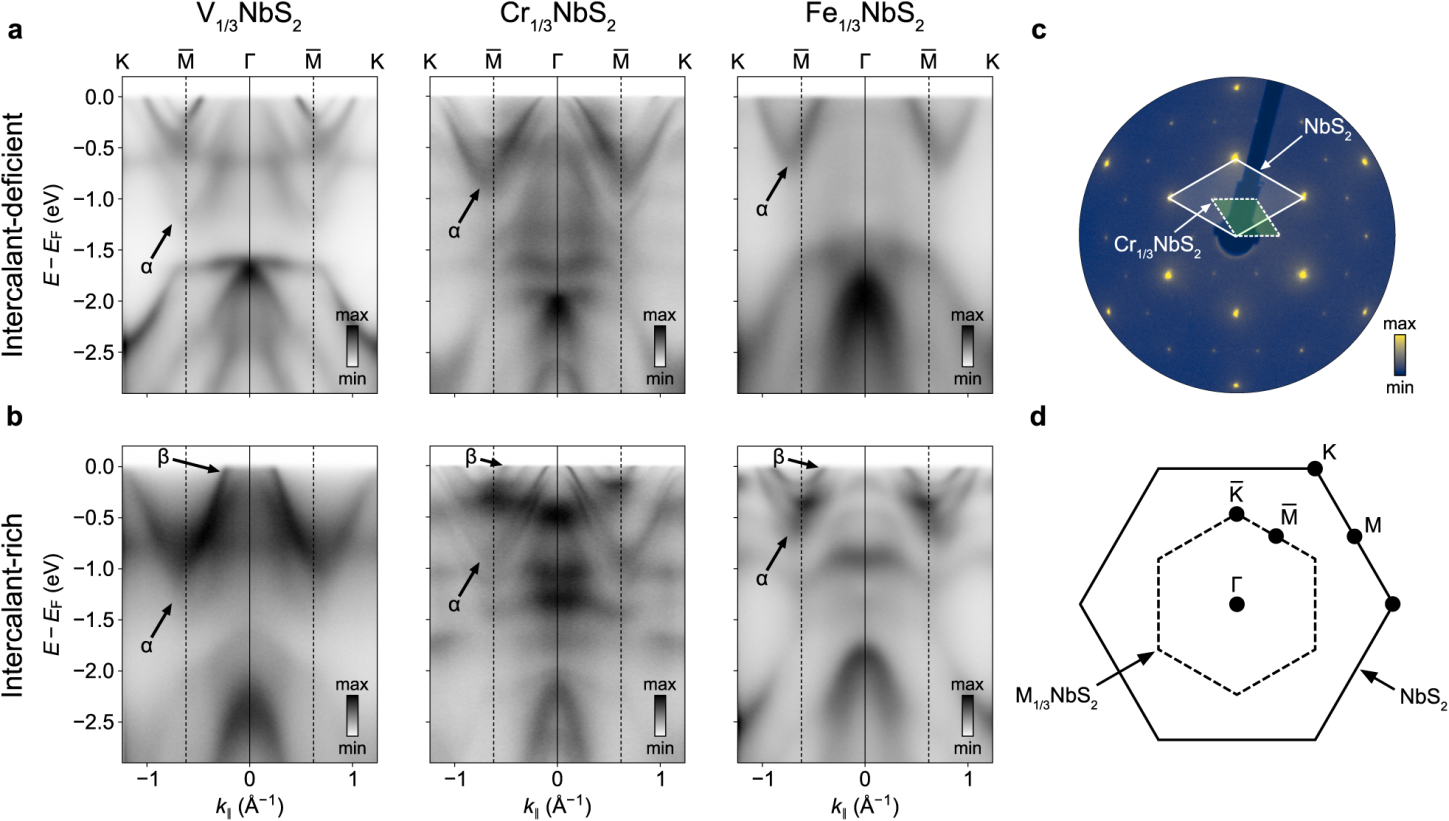
Surface termination-dependent electronic structure of  compounds. (a,b) ARPES dispersions along Γ-K ( eV, LH polarization) of the (a) intercalant-deficient and (b) intercalant-rich surface terminations of  (*T* = 33 K),  (*T* = 20 K), and  (*T* = 38 K). States labeled α and β are bulk- and surface-derived, respectively. (c) LEED image of  measured using a 220 eV electron energy (*T* = 14 K) demonstrating how the intercalants generate the reduced and rotated Brillouin zone shown in (d). The reciprocal space unit cells of the NbS_2_ and  lattices are indicated. (d) Schematic of the Brillouin zones of NbS_2_ and  at . A reduced and rotated Brillouin zone is observed in  compared to NbS_2_, generated by the  superstructure of intercalant atoms. The high symmetry points of the  Brillouin zone are labeled with overbars. Backfolding of some states around the reduced Brillouin zone boundaries is evident in (a,b), indicated by the dashed lines.

Having identified areas of at least dominantly distinct terminations, we turn to the corresponding electronic structures. We summarize these in Figure 3a,b. These measurements were performed along the Γ-K direction of the NbS_2_ lattice surface Brillouin zone. However, as shown in the low-energy electron diffraction (LEED) image and Brillouin zone schematic in Figure 3c,d, the  intercalant superlattice periodicity will give rise to a reduced and rotated Brillouin zone in  compounds. Signatures of this reduced zone can be seen by inspecting the dispersions in Figure 3a,b, where a backfolding of some states around the reduced Brillouin zone boundary ( point, indicated by a dashed line) is evident. In all of the measured surface termination-dependent electronic structures, we observe two manifolds of highly dispersive bands. From comparison to the electronic structure of 2H-NbS_2_,^46,47^ we conclude that the states below ∼1.5 eV are dominantly S-derived, while the dispersive states in the vicinity of the Fermi level are mostly Nb 4*d*-derived.

Detailed interpretation of the electronic structures is, however, made more challenging by the presence of spectral signatures of both surface and bulk states in our measurements, with the former resulting from the distinct surface environment. Comparing the variations seen in the two surface terminations of the compounds, we assign the states labeled α in Figure 3a,b as the bulk Nb-derived conduction bands. These states are located at distinct binding energies for the different intercalant species, pointing to a significant variation in interlayer charge transfer in these compounds. Indeed, in  and , the +3 oxidation states of the intercalants are seen to give rise to more heavily electron-doped Nb-derived α bands, as compared to in  where the Fe intercalants only have a +2 oxidation state. Furthermore, the Nb-derived α band in  can be seen to be less electron-doped than in . Consistent with our ResPES measurements, this points to a deviation away from an ionic-like charge transfer picture in , with stronger hybridizations between intercalant and NbS_2_ layers than in  instead being present, giving rise to a reduced occupied bandwidth.

An additional feature of the low-energy electronic structures is the presence of flatter, and thus more localized, states, which we assign as intercalant-derived. Consistent with our surface termination assignments, these states become more intense on the intercalant-rich surface terminations shown in Figure 3b. The electronic structures presented here thus provide insights into the results of intercalant-specific PDOS obtained from our ResPES measurements. Indeed, in , the lowest binding energy localized V-derived state we observe is positioned relatively far from the Fermi level at ∼0.8 eV (consistent with our ResPES results in Figure 1d) and shows little sign of hybridization with the dispersive NbS_2_-derived states, remaining largely dispersionless. From such an electronic structure, we would expect minimal V-derived spectral weight at the Fermi level, consistent with our ResPES measurements and an RKKY-mediated mechanism of the magnetism in . In contrast, in , localized Cr-derived states are present much closer to the Fermi level, and hybridizations with the itinerant Nb-derived states are evident by spectral weight transfers from Cr- to Nb-derived states. In particular, the Cr-derived state at a binding energy of ∼0.4 eV develops a noticeable dispersion, particularly evident for the Cr-rich surface termination, providing further evidence of the hybridization between localized and itinerant states and leading to Cr-derived weight in the vicinity of the Fermi level, consistent with our ResPES measurements and PDOS calculations in Figure 1d. For the Fe-based compound, localized intercalant-derived states are positioned even closer to the Fermi level. Here, yet stronger hybridizations between intercalant- and Nb-derived states are evident, where the flatter intercalant-derived states are seen to develop considerable dispersion, and hybridization gaps resulting from avoided crossings with the Nb-derived states are observed. This gives rise to the strong Fe-derived spectral weight at the Fermi level shown in Figure 1d. As such, an RKKY picture of magnetism in  and  can be conclusively ruled out, instead pointing towards mechanisms involving substantial hybridizations between intercalant- and Nb-derived states, such as a previously suggested Hund’s coupling.^25^

**Figure 4:**
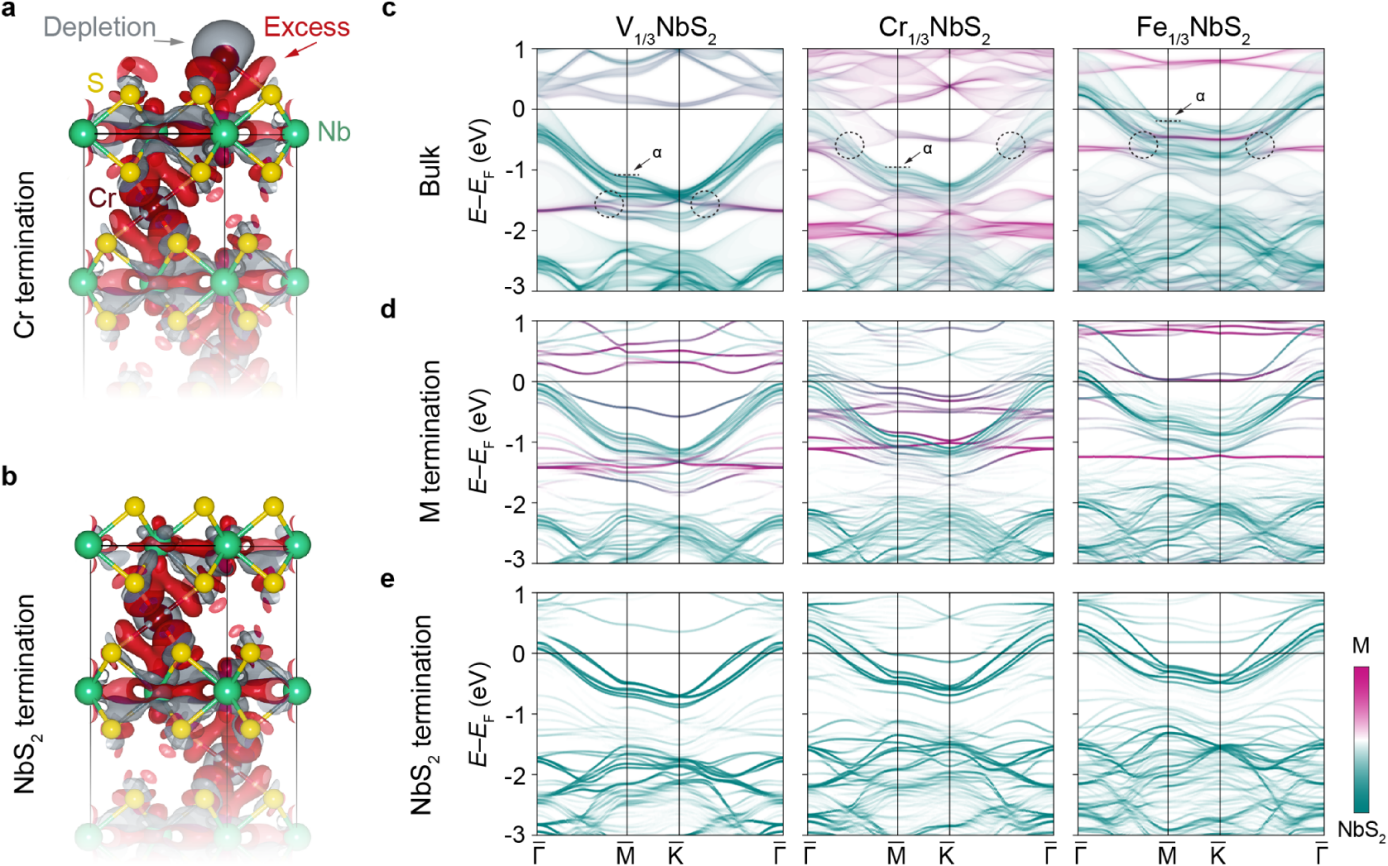
Calculated bulk and surface-termination dependent electronic structure of  compounds. (a,b) Computed excess-depletion charge density distribution for the intermediate case of  for the (a) intercalant and (b) NbS_2_ surface terminations. Here, the excess (depletion) charge is defined as the effective charge added (subtracted) to (from) the pristine NbS_2_ lattice due to Cr intercalation. (c) Site-projected bulk electronic structure of , , and  integrated over all *k*_*z*_ values along a high symmetry path in the reduced Brillouin zone. Here, α denotes the upper edge of the topmost NbS_2_-derived valence band in each compound. The dashed circles indicate the typical regions where orbital mixing between the intercalant- and NbS_2_-derived states leads to a series of hybridization gaps, appearing as abrupt discontinuities in the energy dispersions of the involved states. (d,e) The corresponding surface electronic structures projected onto the (d) intercalant and (e) NbS_2_ terminations.

By comparing the dispersions of the intercalant-deficient surface terminations shown in Figure 3a to the intercalant-rich surface terminations in Figure 3b, a general trend of a more complex near-Fermi level electronic structure with more states visible on the intercalant-rich surface terminations is observed. We label these new dispersive states as β. Similar states have been observed previously in the  class of materials, with their origin being the topic of considerable debate.^25,28,38−40^ Such states are inconsistent with the bulk electronic structure (Figure 4c). Given the strong termination-dependent changes evident here, we thus consider their origin as arising from the polar surfaces of these compounds.

On a pure NbS_2_-exposed surface termination, the absence of an intercalant layer above can be expected to generate hole-doped surface-derived analogues of the bulk NbS_2_ states, as demonstrated in our previous work on .^37^ Indeed, such states can be seen above the α band in Figure 3a for the intercalant-deficient surface termination of . Equivalent states are not clearly observed on the intercalant-deficient surface termination of  and , likely due to a larger presence of disordered intercalants at the surface in these compounds than on the V-based compound, giving rise to a broadening and reduction in spectral weight of any surface states. That such clean NbS_2_-exposed surface terminations are only observed for  is consistent with the weaker bonding between NbS_2_ and intercalant layers discussed above than for the Cr- and Fe-based compounds, resulting in a more natural cleave plane. However, as the intercalant concentration increases, the surface can be expected to become more ordered, leading to the emergence of the visible surface states labeled as β in Figure 3b.

To gain a more comprehensive understanding of these electronic states, we conducted systematic bulk and surface electronic structure calculations. In Figure 4a,b, we present the excess-depletion charge density distributions of  for the intercalant and NbS_2_ surface terminations, respectively. Such a picture is typical for all three compounds studied. The intercalant ions inject a significant charge into the van der Waals gap between neighboring NbS_2_ layers, intensifying interlayer bonding. Crucially, this facilitates effective magnetic exchange interactions between the intercalant sites across the NbS_2_ layers. In contrast, charge density within the NbS_2_ layers experiences partial depletion, indicating that the Nb ion, in the presence of the M intercalant, partially regains its nominal 3+ oxidation state, deviating from the pristine NbS_2_ compound’s 4+ state. At the surface terminations, a noticeable distinction emerges. While the NbS_2_ surface terminations appear to only be minimally affected for the Cr-derived case shown here, the intercalant surface termination accumulates the maximum charge compared to the underlying layers. Consequently, the NbS_2_ states on the M-terminated side are expected to be maximally electron-doped, leading to a downward shift within the overall energy spectrum. Furthermore, the intercalant surface termination ensures a significant contribution of the intercalant 3*d* orbitals to the electronic structure.

Despite the apparent similarities of the different  compounds in real space, a profound disparity arises in their momentum space. In Figure 4c and Supplementary Figure 7, we present a comparison of the calculated site-projected bulk electronic structures for , , and . Moving from V to Fe, we can see escalating alterations in the electronic dispersions of the host NbS_2_ bands due to the influence of the intercalant 3*d* orbitals. In , V-derived states primarily contribute as nonbonding flat bands residing at a binding energy of ∼1.5 eV, slightly overlapping with the lower edge of the NbS_2_-derived valence bands. Consequently, the resulting electronic structure bears a resemblance to that of pristine NbS_2_. Consistent with our above discussions, this suggests that any magnetic alignment between V ions can only be stabilized through indirect exchange coupling processes, such as the RKKY interaction, facilitated by charge carriers at the Fermi level. In contrast, the Cr 3*d* orbitals in  have a higher filling number compared to the V case, and thus exhibit a more substantial presence at and below the Fermi level, effectively hybridizing with the topmost NbS_2_-derived bands and giving rise to additional dispersive bands over a wide energy range. This implies a more inherently itinerant form of magnetism in , a conclusion supported by our ResPES and ARPES measurements. The situation becomes even more pivotal in , where the Fe states significantly modify the electronic structure away from the picture of pristine NbS_2_. The preference of Fe to maintain its  state allows its 3*d* orbitals to reside at energies considerably below the Fermi level, enabling robust hybridization with both S 3*p* and Nb 4*d* states (Supplementary Notes 4 and 5 and the associated figures present a comparative analysis of intercalant hybridization and its effect on host electronic states in ). This leads to a broadened bandwidth for the entire bulk valence continuum and an upward shift of the original topmost NbS_2_ bands, as is evident in Figure 4c.

Figure 4d,e shows the surface electronic structures for the intercalant and NbS_2_ surface terminations, respectively. Moving from V to Fe, we can again see a noticeable overall shift of the bands toward lower binding energies for both terminations. This shift, as explained above, is attributed to the significant enhancement of hybridizations between intercalant 3*d* orbitals and host NbS_2_ states in  and  compared to , with  exhibiting the most pronounced electronic structure modification. Comparison of the two terminations illustrates a consistently deeper energy spectrum for the intercalant surface terminations across all three compounds. This finding aligns with our excess-depletion charge density calculations: while the subsurface NbS_2_ layer can now be expected to retain close to its bulk-like configuration, with charge transfer from intercalant layers above and below, the surface intercalant layer will now become electron-doped due to the absence of a NbS_2_ layer above, giving rise to the β states observed experimentally. For the NbS_2_ surface terminations (Figure 4e), the intercalants here lack direct contributions to the energy bands. Nevertheless, they indirectly influence the details of the band dispersions, particularly at and near the Fermi level, owing to their spatial and energetic proximities to these bands. Thus, these surface terminations serve as an ideal medium for creating magnetically controllable carriers with rich valleytronic properties.^37^

In contrast, for the intercalant surface terminations shown in Figure 4d, a distinct disparity in the formation of intercalant-derived bands near the Fermi level becomes evident. While  displays a single flat intercalant-dominated band in the occupied electronic structure, at a binding energy of ∼0.5 eV,  instead possesses a ladder of such bands spanning a ∼1 eV energy window below the Fermi level. In , a well-localized flat band is located at 1 eV binding energy, while additional significant intercalant-derived spectral weight is visible in the vicinity of the Fermi level and also at deeper binding energy (Supplementary Figure 7). The bands sitting just at the Fermi level are likely slightly occupied in the experimental measurements, leading to the complex electronic structure near the Fermi level observed in Figure 3b. These evidently have a mixed Nb–Fe character, enabling their dispersion down to the Fermi level, and their population in the electron-doped surface layer.

While these new β states are derived from the intercalant-terminated surfaces, their mixed atomic character is clear from their significant dispersion, evident in both our measured dispersions and Fermi maps (see Supplementary Figure 3). Indeed, their dispersive nature has led to some challenges in assigning these states. In particular, in , the presence of the β bands at the Fermi level has been of significant attention, with splittings in these attributed to an exchange splitting in the low-energy electronic structure of .^25,48^ However, as discussed in Supplementary Note 3, with the new understanding of surface- and bulk-derived states advanced here, we can now rule out the possibility that the observed temperature-dependent changes are related to any resolvable exchange splitting of itinerant Nb-derived states.

## 4 Conclusions

Our measurements on , , and , and corresponding DFT calculations, have demonstrated a pronounced and element-specific role of the intercalants on the electronic structure of metal-intercalated TMDs. In all three compounds, marked differences were observed in the electronic structure as compared to those of 2H-NbS_2_. These include electron doping of bands from interlayer charge transfers and the backfolding of bands around the reduced Brillouin zone boundaries that are generated by the intercalant superlattices. Furthermore, shifts in the chemical potential that arise from self-doping at polar surfaces led to distinct surface terminations in these compounds. It was additionally shown that the choice of intercalant species leads to varying amounts of hybridizations between localized intercalant- and itinerant Nb-derived states, resulting in nontrivial differences between the electronic structure of these compounds. Indeed, the minimal hybridizations in  lead to a scenario where the effect of the intercalant is well-described by the ionic-like charge transfers of a rigid band shift picture, in turn facilitating RKKY-mediated magnetic interactions. In contrast, stronger interlayer hybridizations in  and  mean that the localized intercalant-derived states are no longer weakly coupled to the itinerant Nb-derived states. As such, an RKKY-like exchange mechanism is insufficient to describe the magnetic interactions in these two compounds, motivating further investigations into the origin of their magnetic order. Furthermore, we provided an explanation for the origin of the anomalous states frequently reported at the Fermi level in the  compounds, as the result of hybridizations between electron-doped surface intercalant-derived and subsurface NbS_2_-derived states. This assignment allowed us to rule out the possibility of a previously reported exchange splitting of the Nb 4*d*-derived conduction bands at the surface of . Together, our combined experimental and theoretical study has identified how systematic changes in the orbital filling and ionicity of the intercalants in  contribute to the creation of magnetically rich surfaces with distinct electronic properties, opening new prospects for tuning these *via* materials design.

## Data Availability

The research data supporting this publication can be accessed at 10.17630/295fc754-5305-461c-9640-557cd304de81.^49^

## Supporting Information Available

Supplementary notes providing additional discussion of (1) spatial mapping photoemission data analysis, (2) Fermi surface measurements, (3) temperature-dependent ARPES measurements of , (4) calculated densities of states, and (5) chemical trends in the degree of electronic hybridization. Supplementary figures of (1,2) spatial mapping ARPES data, (3) Fermi surface measurements, (4) temperature-dependent ARPES, (5) density of states calculations, (6) resonant photoemission measurements, (7,8) calculated bulk electronic structures and spin densities, and (9) magnetic characterization (PDF)
