# Supplementary material for: Chemical Trends of the Bulk and Surface Termination-Dependent Electronic Structure of Metal-Intercalated Transition Metal Dichalcogenides
Source: Chem Mater. 2024 Jul 23;36(15):7117–26. doi: 10.1021/acs.chemmater.4c00824 (PMC11325556; doi:10.1021/acs.chemmater.4c00824)
Supplement: Supplementary file 1 — cm4c00824_si_001.pdf [file cm4c00824_si_001.pdf]

Supplementary material: Chemical trends of the bulk and surface termination-dependent electronic structure of metal-intercalated transition metal dichalcogenides

Brendan Edwards,[1] Darius-A. Deaconu,[2] Phil A. E. Murgatroyd,[1] Sebastian Buchberger,[1,3] Tommaso Antonelli,[1] Daniel Halliday,[1,4] Gesa-Roxanne Siemann,[1] Andela Zivanovic,[1,3] Liam Trzaska,[1] Akhil Rajan,[1] Edgar Abarca Morales,[1,3] Daniel A. Mayoh,[5] Amelia E. Hall,[5] Rodion V. Belosludov,[6] Matthew D. Watson,[4] Timur K. Kim,[4] Deepnarayan Biswas,[4] Tien-Lin Lee,[4] Craig M. Polley,[7] Dina Carbone,[7] Mats Leandersson,[7] Geetha Balakrishnan,[5] Mohammad Saeed Bahramy,[2,*] and Phil D. C. King[1,†]

[1]*SUPA, School of Physics and Astronomy, University of St Andrews, St Andrews KY16 9SS, UK*
[2]*Department of Physics and Astronomy, University of Manchester, Oxford Road, Manchester M13 9PL, UK*
[3]*Max Planck Institute for Chemical Physics of Solids, Nöthnitzer Strasse 40, 01187 Dresden, Germany*
[4]*Diamond Light Source Ltd, Harwell Science and Innovation Campus, Didcot OX11 0DE, United Kingdom*
[5]*Department of Physics, University of Warwick, Coventry CV4 7AL, United Kingdom*
[6]*Institute for Materials Research, Tohoku University, Sendai 980-08577 Japan*
[7]*MAX IV Laboratory, Lund University, P. O. Box 118, 221 00 Lund, Sweden*

(Dated: July 9, 2024)

SUPPLEMENTARY NOTE 1: REGION OF INTEREST ANALYSIS OF ARPES SPATIAL MAPPING

We show in Supplementary Fig.1 the analysis of our spatially-resolved ARPES measurements of $V_{1/3}NbS_2$, $Cr_{1/3}NbS_2$ and $Fe_{1/3}NbS_2$. These measurements were performed over the same spatial region as the Nb $4p$ core level spatial maps shown in Fig. 2(b) of the main text. The spatial maps in Supplementary Fig.1(a) were obtained by integrating the spectral weight within a region of interests indicated by dashed lines in Supplementary Fig.1(b,c) which contain intercalant-derived flat-band states. As discussed in the main text, such states become enhanced on the intercalant-rich surface termination, and thus region of high (yellow) and low (blue) intensity regions correspond to the intercalant-rich and intercalant-deficient surface terminations, respectively.

We note that, in particular for the Cr- and Fe-based systems, it is likely that we do not have pure terminations within our probing beam. Indeed, observations in Ref. 38 of the main text suggest variations on a length scale of ~ 200 nm, substantially below the spatial scale of our probing light spot. This will lead to averaging in our data, however, we can still track the dominant changes in terminating surface. We show, for example, in Supplementary Fig. 2 a line scan between two different dominant regions of $Cr_{1/3}NbS_2$. This shows a gradual evolution, suggesting a slow change from a region dominated by NbS_2 termination, and one with a dominant Cr-termination.

SUPPLEMENTARY NOTE 2: ANALYSIS OF FERMI MAP MEASUREMENTS

We show in Supplementary Fig.3 the analysis of our Fermi surface measurements of the intercalant-rich surface terminations of $V_{1/3}NbS_2$, $Cr_{1/3}NbS_2$ and $Fe_{1/3}NbS_2$. We present in Supplementary Figs.3(b-e) cuts through the Brillouin zone taken parallel to the Γ-K high symmetry direction, as indicated by the dotted lines in the Fermi surfaces in Supplementary Fig.3(a). Here, it can be observed that there are clear dispersions of the β bands defined in the main text, with similar form as the bulk-like Nb $4d$-derived α bands, pointing to a non-negligible Nb character. Nonetheless, contrasting spectral weights of the α and β bands result from a substantial intercalant character of the β bands (see Fig. 4 of the main text).

SUPPLEMENTARY NOTE 3: ANALYSIS OF THE TEMPERATURE DEPENDENCE OF THE ELECTRONIC STRUCTURE OF $Cr_{1/3}NbS_2$

We show in Supplementary Figs. 4(a,b) additional measurements of the band dispersions of the Cr-rich surface terminations using linear horizontal (LH) and linear vertical (LV) polarisations, respectively. To aid discussion, the bulk-derived α and surface-derived β bands are labelled. A backfolding of these bands around the reduced Brillouin zone boundary additionally leads to the $\alpha^{\rm bf}$ and $\beta^{\rm bf}$ replica bands. Previously, a temperature-dependent evolution of band shifts in $Cr_{1/3}NbS_2$ has been attributed to a magnetic exchange splitting of the itinerant states [1, 2]. To explore this further, we show new temperature-dependent measurements for the Cr-rich surface in Supplementary Figs. 4(c-e). The orange arrows trace the approximate evolution with temperature of the α, $\beta^{\rm bf}$ and β bands identified above. As temperature increases, the α band shifts upwards to lower binding energies, while the β and $\beta^{\rm bf}$ shift in the opposite direction to deeper binding energies. This results in a reduced separation between the α and β bands, as depicted in Supplementary Fig. 4(h). At high temperatures, the α and $\beta^{\rm bf}$ band become indistinguishable, as can be seen clearly in the temperature-dependent Fermi level MDCs shown in Supplementary Fig. 4(f) where the low-temperature three-peak structure develops into a broad two-peak structure above the ~ 125 K magnetic transition. However, the broad peak consisting of the α and $\beta^{\rm bf}$ bands remains asymmetric at high temperature, indicative that a small separation between the bands persists. This is confirmed in the temperature dependence of the α, $\beta^{\rm bf}$ and β bands peak positions shown in Supplementary Fig. 4(g), obtained by fitting the Fermi level MDCs. Additionally, it can be seen that the β and $\beta^{\rm bf}$ bands exhibit near-identical temperature-dependent shifts, providing further evidence that they are indeed backfolded copies of each other. While the α and $\beta^{\rm bf}$ bands approach each other as temperature is increased, this cannot be assigned to an exchange splitting, as suggested previously [1, 2], due to the distinct bulk and surface origins of the two states, respectively. Instead, we propose that the temperature-dependent binding energy shifts observed here reflect varying hybridisations with local moment Cr-derived states. While experimental resolution means we cannot rule out the possibility of an unresolved exchange splitting of the α or β bands, any such splittings would be very small, on the scale of a few meV at most.

* m.saeed.bahramy@manchester.ac.uk
† pdk6@st-andrews.ac.uk

SUPPLEMENTARY NOTE 4: TOTAL AND INTERCALANT-PROJECTED DENSITY OF STATES

Supplementary Fig. 5 illustrates the calculated total and intercalant-projected density of states for the bulk $M_{1/3}NbS_2$ compounds. In $V_{1/3}NbS_2$, the V ions predominantly contribute as non-bonding states, resulting in a sharp Van Hove singularity peaking at ~ 1.5 eV below the Fermi level. Similarly, in $Cr_{1/3}NbS_2$, a single Van Hove peak originating from Cr $3d$ orbitals is seen below the Fermi level, albeit at a lower energy compared to $V_{1/3}NbS_2$. This peak broadens over a larger energy range, indicating enhanced hybridization between Cr $3d$ orbitals and NbS_2 states. In contrast, $Fe_{1/3}NbS_2$ exhibits a markedly different behavior. Here, the Fe states manifest in two distinct ways: one as a localized peak slightly below the Fermi level and the other as a bonding-anti-bonding distribution spanning over a broad energy window from 2 eV to 6 eV below the Fermi level. Notably, there is a pronounced alteration in the total density of states in the latter region compared to the other two compounds, suggesting significant hybridization between Fe $3d$ orbitals and their NbS_2 host states. Consequently, $Fe_{1/3}NbS_2$ is anticipated to demonstrate a robust itinerant-type magnetic exchange coupling between Fe ions. Furthermore, the appearance of a peak near the Fermi level indicates that Fe $3d$ electrons may effectively contribute to charge transport and low-energy excitations in $Fe_{1/3}NbS_2$. The findings from our calculations are supported by our resPES measurements shown in the main text and in Supplementary Fig. 6.

SUPPLEMENTARY NOTE 5: EVOLUTION OF THE ELECTRONIC HYBRIDISATIONS

To provide further insight into the interplay between the intercalant ions and NbS_2 layers, we show in Supplementary Fig. 7 the computed site-projected bulk electronic structures over a wide energy range around the Fermi level for NbS_2, $V_{1/3}NbS_2$, $Cr_{1/3}NbS_2$, and $Fe_{1/3}NbS_2$. Focusing on the α bands, it is evident that they reside at significantly lower energies in all three $M_{1/3}NbS_2$ compounds compared to their counterparts in NbS_2. This observation confirms an overall charge transfer from the intercalant to the host NbS_2 layers. However, moving from V to Fe, the α bands exhibit a consistent upward shift in energy due to the increased hybridization between NbS_2- and intercalant-derived states. This enhancement in hybridisation is illustrated by highlighted boxes in Supplementary Figs. 7(b-d). In $V_{1/3}NbS_2$, shown in Supplementary Fig. 7(b), V states are notably absent near the Fermi level, primarily manifesting as non-bonding flat bands located at binding energies of ~ 1.5 eV with only a slight overlap with the lower edge of the NbS_2-derived valence bands. Consequently, the resultant electronic structure closely resembles that of pristine NbS_2, shown in Supplementary Fig. 7(a), with an additional energy shift due to charge transfer between the V and NbS_2 states. In contrast, as can be seen in Supplementary Fig. 7(c), $Cr_{1/3}NbS_2$ exhibits a distinct scenario where Cr $3d$ orbitals appear dominantly over a wider energy range near the Fermi level, forming effective hybridisations with the NbS_2-derived bands across a broad energy range. This interaction gives rise to multiple additional dispersive bands at and near the Fermi level. This situation is further intensified in $Fe_{1/3}NbS_2$, as can be seen in Supplementary Fig. 7(d). Here, there is a significant portion of Fe $3d$ states concentrated at binding energies of ~ 4 eV, allowing robust hybridisation with S $3p$ and Nb $4d$ bands. This results in a discernible modification of the bandwidth and electronic dispersions across the entire valence band continuum in this system. We attribute this phenomenon to the strong tendency of Fe to maintain its $3d^6$ state, facilitating a more effective alignment of its $3d$ orbitals with the occupied bands in the host NbS_2 layers and thereby promoting robust hybridisations.

Similar conclusions can be obtained by inspecting the calculated spin density, shown in Supplementary Fig. 8. The spin density in $V_{1/3}NbS_2$ is essentially localized around the V ions, with a minor presence over its adjacent S and Nb ions. On the other hand, in the other two compounds, particularly $Fe_{1/3}NbS_2$, the spin density spreads out significantly over the entire crystal, resulting in a more pronounced contribution from the NbS_2 layer. This further confirms the itinerant nature of exchange coupling in these latter systems, as opposed to the weak RKKY mechanism in $V_{1/3}NbS_2$, where local V moments are effectively decoupled from one another and can only exchange their magnetic state through the carriers.

---

[1] Sirica, N. *et al.* The nature of ferromagnetism in the chiral helimagnet $Cr_{1/3}NbS_2$. *Commun. Phys.* **3**, 65 (2020).

[2] Qin, N. *et al.* Persistent exchange splitting in the chiral helimagnet $Cr_{1/3}NbS_2$. *Phys. Rev. B* **106**, 035129 (2022).

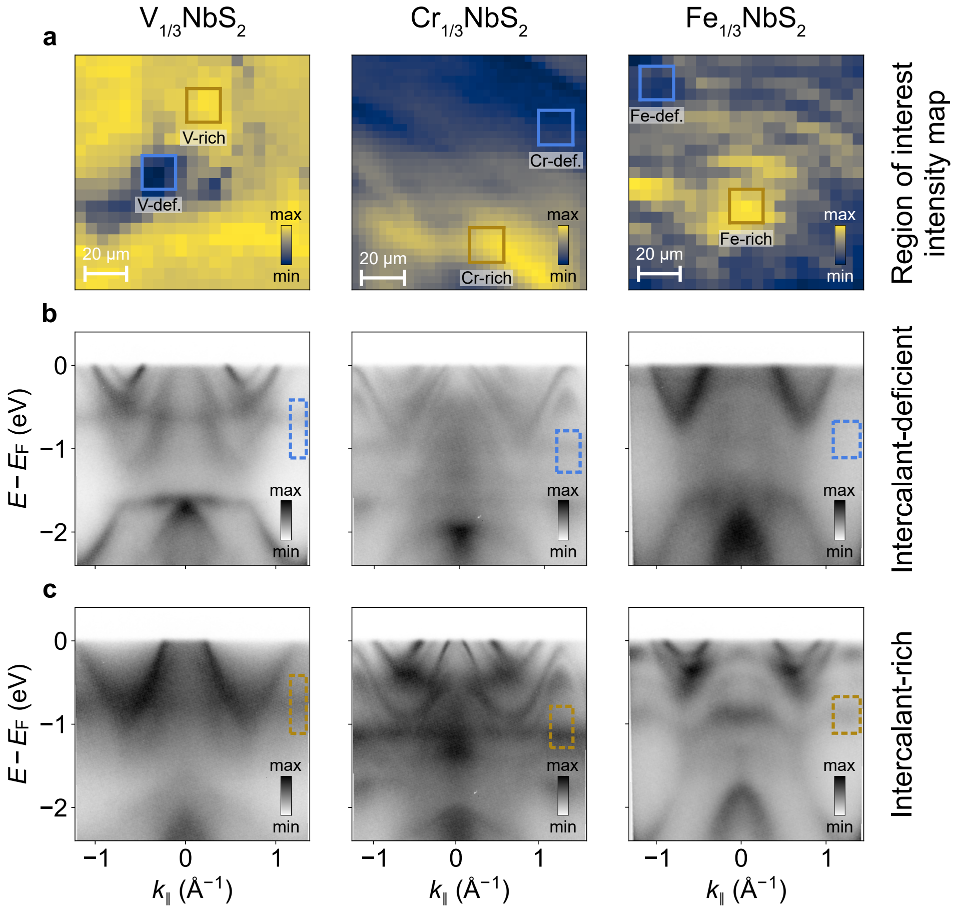

Supplementary Fig. 1. ARPES spatial mapping of $M_{1/3}NbS_2$ compounds. (a) Spatial maps of $V_{1/3}NbS_2$, $Cr_{1/3}NbS_2$ and $Fe_{1/3}NbS_2$ displaying the integrated spectral weight within the region of interests indicated by dashed lines in (b) and (c), corresponding to intercalant-derived states. High (yellow) and low (blue) intensity regions correspond to the intercalant-rich and intercalant-deficient surface terminations, respectively. (b,c) Representative dispersions along K-Γ-K (LH polarisation) of the (b) intercalant-deficient and (c) intercalant-rich surface terminations of $V_{1/3}NbS_2$ (T = 33 K, $h\nu$ = 79 eV), $Cr_{1/3}NbS_2$ (T = 35 K, $h\nu$ = 100 eV) and $Fe_{1/3}NbS_2$ (T = 36 K, $h\nu$ = 80 eV), extracted from the regions of spatial mapping data indicated in (a).

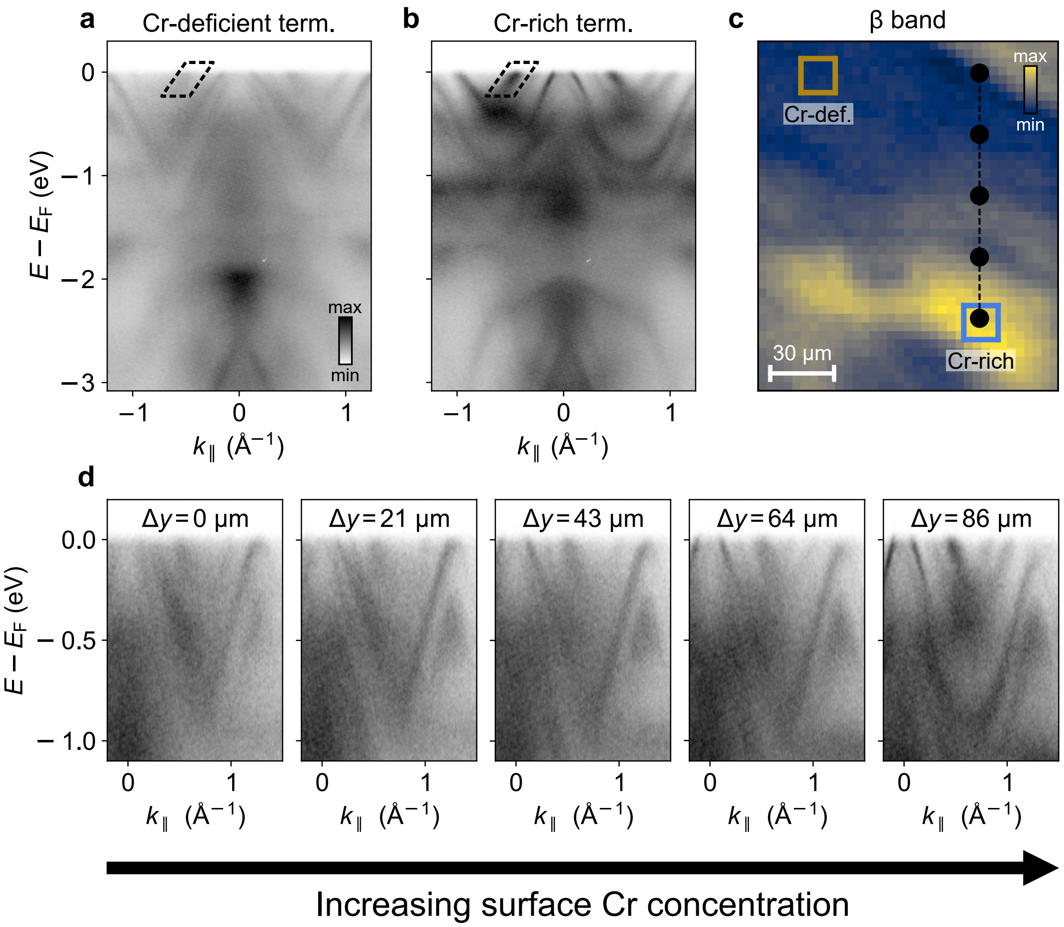

Supplementary Fig. 2. Evolution of dominant surface terminations in spatially-dependent ARPES measurements of $Cr_{1/3}NbS_2$. (a,b) Dispersions along K-Γ-K of the (a) Cr-deficient and (b) Cr-rich surface terminations ($T = 35$ K, $h\nu = 100$ eV, LH polarisation), extracted from the regions of spatial mapping data indicated in (c). (c) Spatial map displaying the integrated spectral weight within the region of interests indicated by dashed lines in (a) and (b). (d) Dispersions extracted from the positions indicated by black markers in the spatial map in (c). Δy represents the position along the dashed line in the spatial map in (c) at which the dispersion was extracted, where $\Delta y = 0$ corresponds to the Cr-deficient surface termination. Thus, increasing Δy correlates with increasing surface Cr concentration.

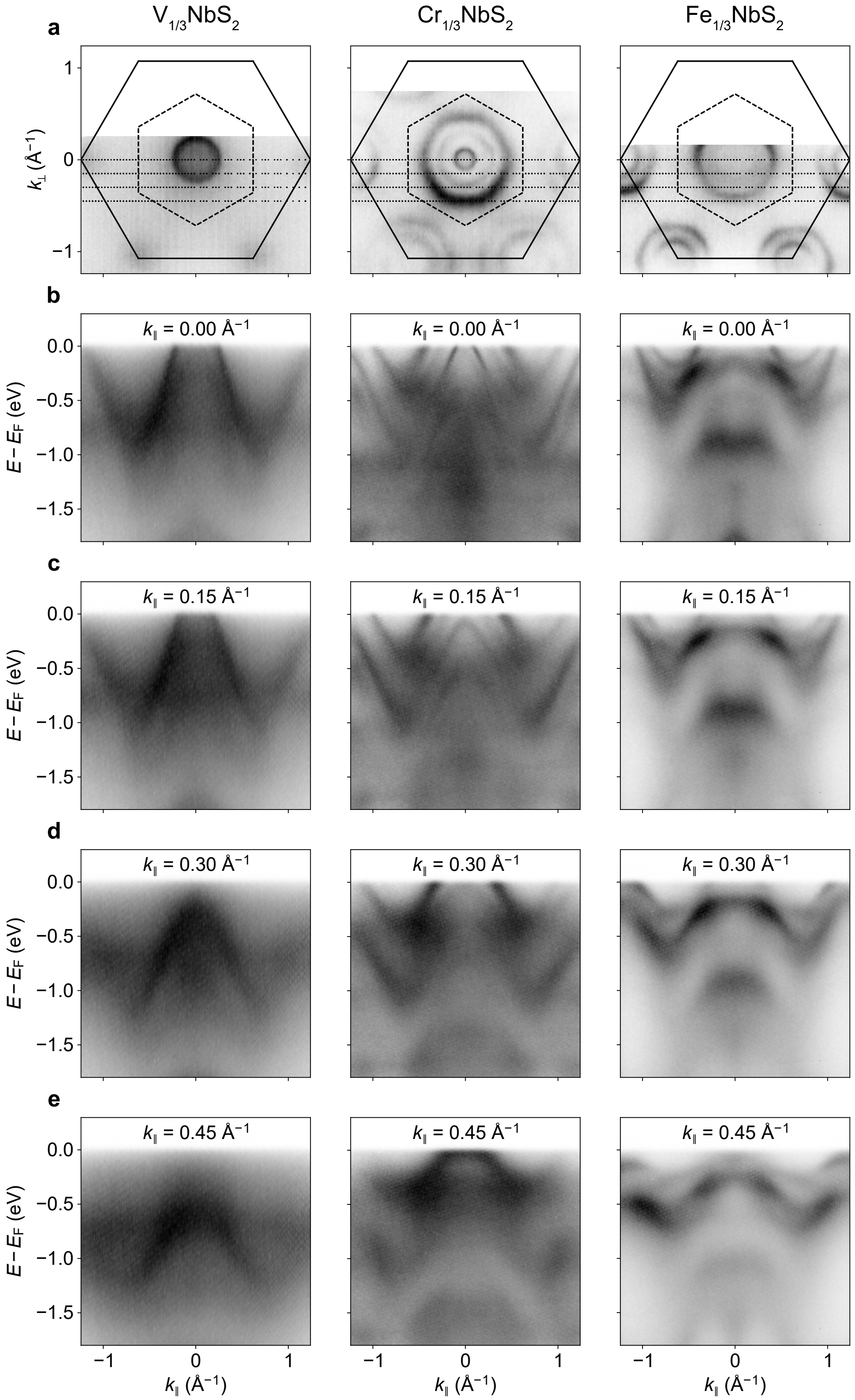

Supplementary Fig. 3. Fermi map analysis of the intercalant-rich surface terminations of $M_{1/3}NbS_2$ compounds. (a) Fermi surfaces (LH polarisation) of $V_{1/3}NbS_2$ (T = 35 K, $h\nu$ = 79 eV), $Cr_{1/3}NbS_2$ (T = 35 K, $h\nu$ = 100 eV) and $Fe_{1/3}NbS_2$ (T = 34 K, $h\nu$ = 79 eV). The solid hexagons indicate the NbS_2 Brillouin zone, whereas the dashed hexagons indicate the reduced $M_{1/3}NbS_2$ Brillouin zone. (b-d) Dispersions extracted along the cuts through the Fermi maps indicated by the dotted lines in (a) at (b) $k_\perp = 0.00$ Å^{-1}, (c) $k_\perp = 0.15$ Å^{-1}, (d) $k_\perp = 0.30$ Å^{-1} and (e) $k_\perp = 0.45$ Å^{-1}. Here, $k_\perp$ indicates the momentum along the in-plane direction perpendicular to the analyser entrance slit and $k_\parallel$ is along the analyser entrance slit.

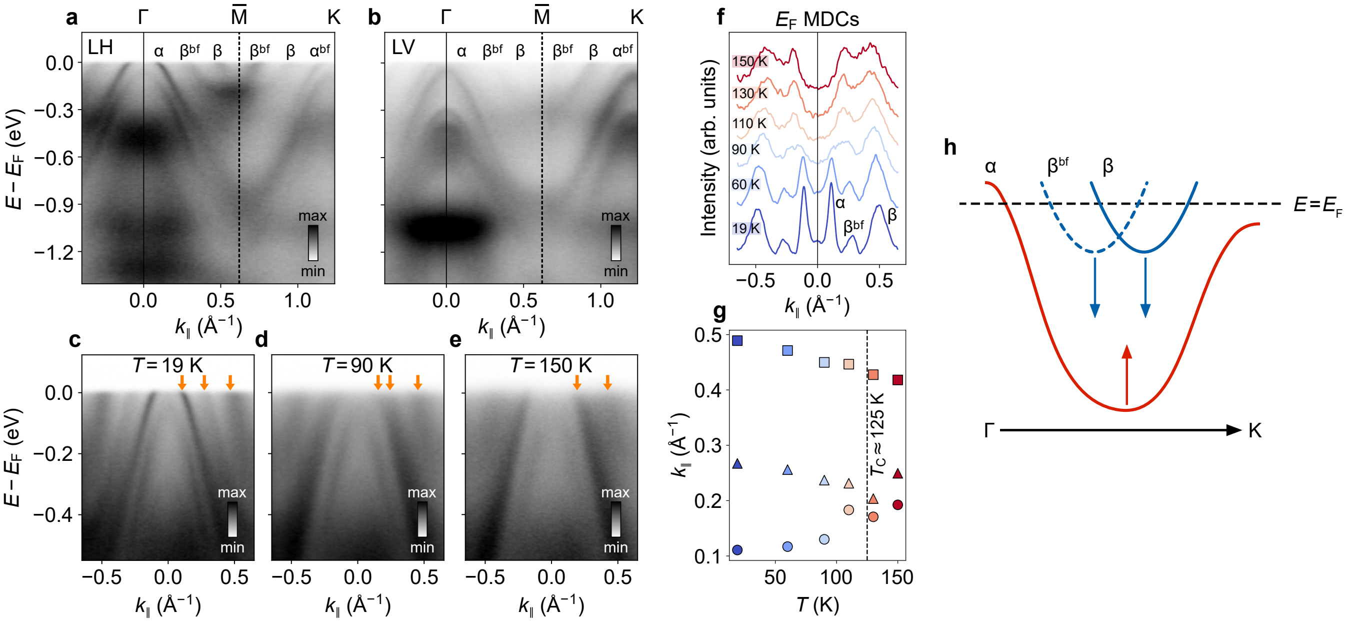

Supplementary Fig. 4. Temperature dependence of the Cr-rich surface termination electronic structure of $Cr_{1/3}NbS_2$. (a,b) Dispersions along Γ-K (T = 20 K, $h\nu$ = 79 eV), measured using (a) LH and (b) LV polarised light. The bulk-derived α and surface-derived β bands are indicated, along with their backfolded copies, α^{bf} and β^{bf}. (c-e) Dispersions along Γ-K ($h\nu$ = 100 eV, LH polarisation) measured at (c) 19 K, (d) 90 K, (e) 150 K. The orange arrows track the approximate peak positions at the Fermi level of the α, β^{bf} and β bands. (f) Temperature-dependent Fermi level MDCs. (g) Temperature-dependent peak positions of the α, β^{bf} and β bands, extracted from fits to the Fermi level MDCs in (f). (h) Schematic of the evolution of the α and β bands as temperature is increased through the $Cr_{1/3}NbS_2$ magnetic transition. The red and blue arrows indicate the direction of the binding energy shifts observed with increasing temperature for the α and β bands, respectively.

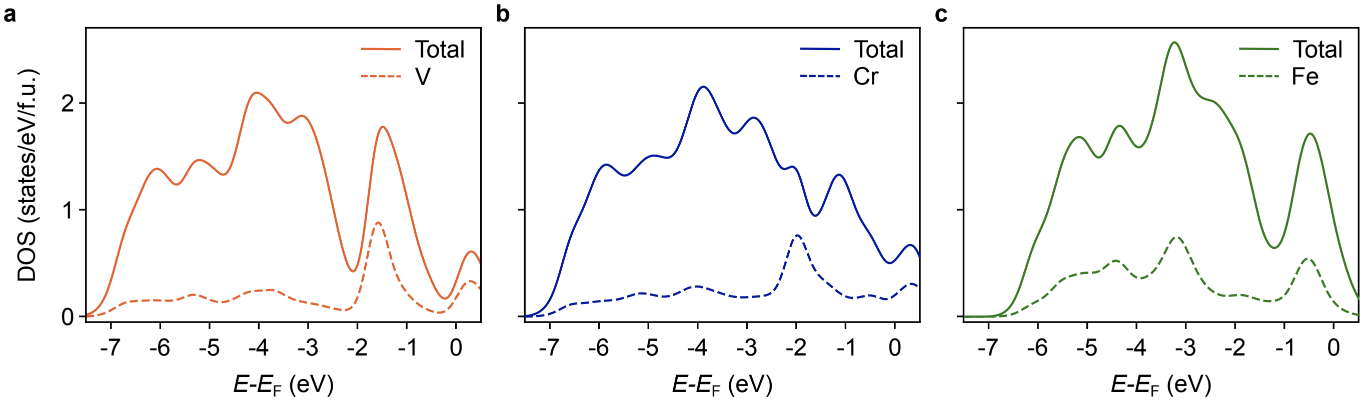

Supplementary Fig. 5. Comparison of the density of states of $M_{1/3}NbS_2$ compounds. (a-c) The total and intercalant-projected density of states calculated for (a) $V_{1/3}NbS_2$, (b) $Cr_{1/3}NbS_2$ and (c) $Fe_{1/3}NbS_2$.

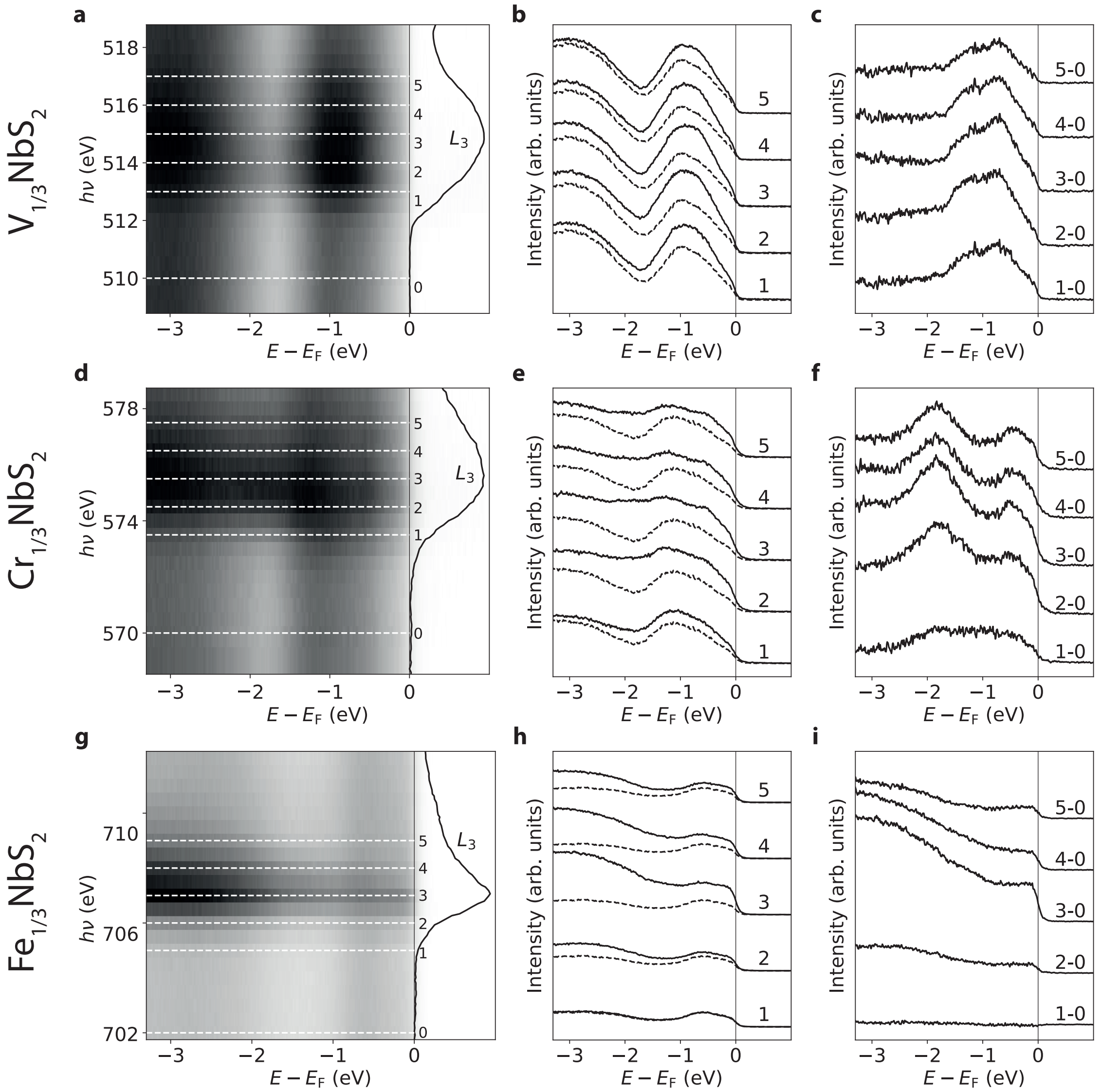

Supplementary Fig. 6. Intercalant-derived spectral weight near the Fermi level of $M_{1/3}NbS_2$ compounds. (a-i) ResPES measurements of (a-c) $V_{1/3}NbS_2$, (d-f) $Cr_{1/3}NbS_2$ and (g-i) $Fe_{1/3}NbS_2$. (a,d,g) k-space-integrated spectra measured across the intercalant L_3 absorption edges (shown in the XAS measurements on the right of the panels), reproduced from the measurements shown in Fig. 1c of the main text. (b,e,h) Comparison of measurements at the select photon energies indicated in (a,d,g) to the off-resonance spectra, shown as a dashed lines. (c,f,i) Corresponding difference spectra between the on-and off-resonance spectra. The ”3-0” spectra are the difference between the spectra taken with the photon energy tuned to the peak of the L_3 XAS edges and the off-resonance spectra, as shown in Fig. 1d of the main text.

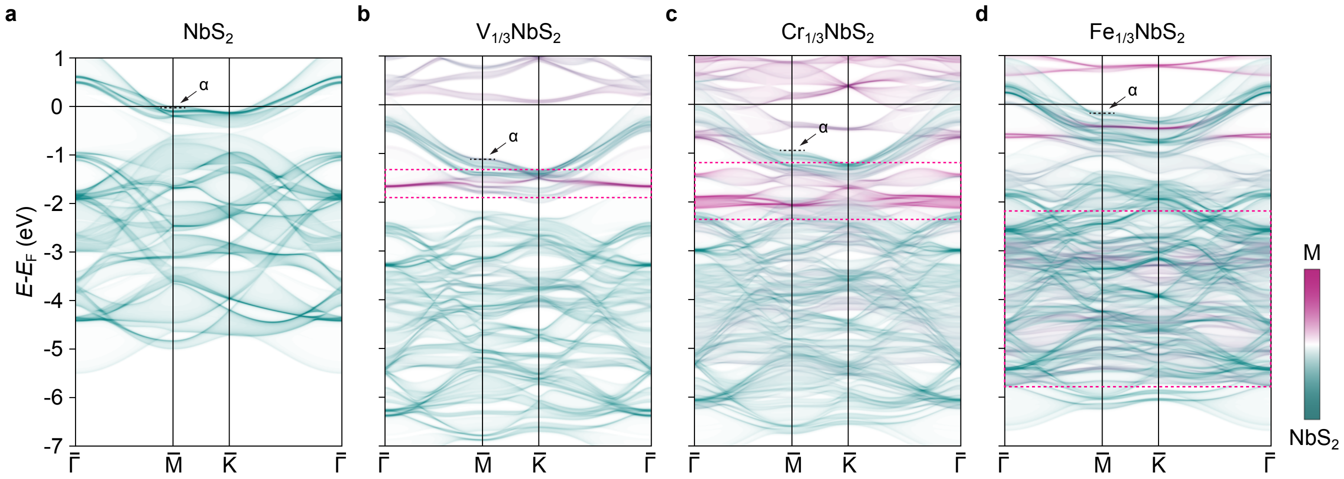

Supplementary Fig. 7. Comparison between the calculated bulk electronic structures of NbS_2 and $M_{1/3}NbS_2$ compounds. (a-d) Site-projected electronic structures of (a) NbS_2, (b) $V_{1/3}NbS_2$, (c) $Cr_{1/3}NbS_2$ and (d) $Fe_{1/3}NbS_2$ integrated over all k_z values along a high symmetry path in the reduced Brillouin zone, with projected site character. Here, α denotes the upper edge of the topmost NbS_2-derived valence band. The highlighted boxes in (b-d) indicate the regions exhibiting the most effective hybridisation between NbS_2- and intercalant-derived states.

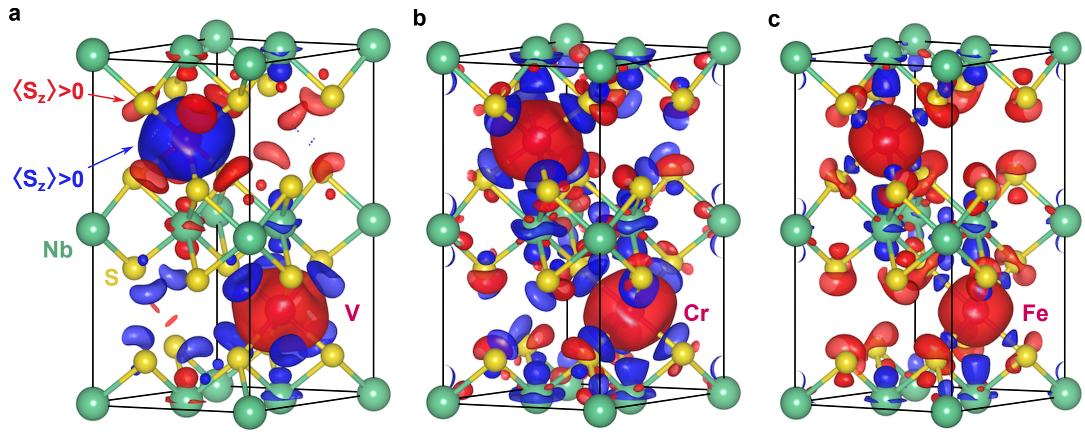

Supplementary Fig. 8. Comparison of the bulk spin densities of $M_{1/3}NbS_2$ compounds. (a-c) The DFT calculated spin densities of (a) $V_{1/3}NbS_2$, (b) $Cr_{1/3}NbS_2$ and (c) $Fe_{1/3}NbS_2$.

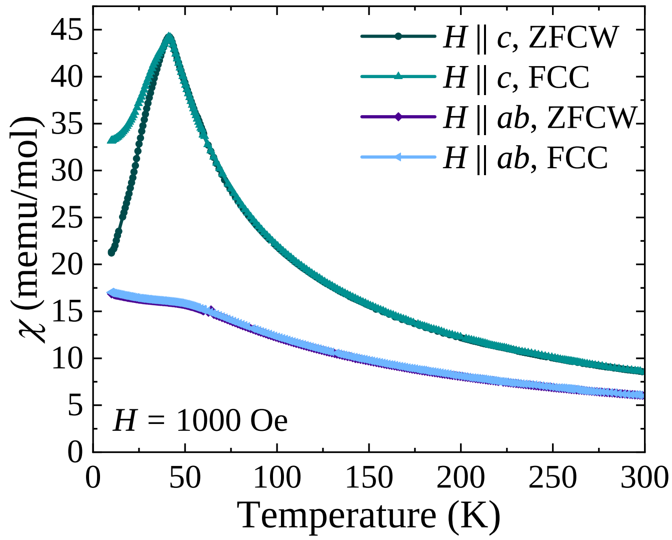

Supplementary Fig. 9. Magnetic susceptibility measurements of $Fe_{1/3}NbS_2$. The presence of the expected magnetic transition at $\sim$ 45 K provides a reliable indicator of the anticipated stoichiometry, given the compound exhibits an extreme sensitivity to the precise composition.
